# Uremic Toxins and Lipases in Haemodialysis: A Process of Repeated Metabolic Starvation

**DOI:** 10.3390/toxins6051505

**Published:** 2014-04-30

**Authors:** Bernd Stegmayr

**Affiliations:** Department of Public Health and Clinical Medicine, University of Umeå, Umeå SE-901 87, Sweden; E-Mail: bernd.stegmayr@medicin.umu.se

**Keywords:** hepatic lipase, lipoprotein lipase, haemodialysis, peritoneal dialysis, malnutrition

## Abstract

Severe kidney disease results in retention of uremic toxins that inhibit key enzymes for lipid breakdown such as lipoprotein lipase (LPL) and hepatic lipase (HL). For patients in haemodialysis (HD) and peritoneal dialysis (PD) the LPL activity is only about half of that of age and gender matched controls. Angiopoietin, like protein 3 and 4, accumulate in the uremic patients. These factors, therefore, can be considered as uremic toxins. In animal experiments it has been shown that these factors inhibit the LPL activity. To avoid clotting of the dialysis circuit during HD, anticoagulation such as heparin or low molecular weight heparin are added to the patient. Such administration will cause a prompt release of the LPL and HL from its binding sites at the endothelial surface. The liver rapidly degrades the release plasma compound of LPL and HL. This results in a lack of enzyme to degrade triglycerides during the later part of the HD and for another 3–4 h. PD patients have a similar baseline level of lipases but are not exposed to the negative effect of anticoagulation.

## 1. Introduction

Haemodialysis patients have a reduced survival compared to age matched persons [1], and the increased risk for morbidity and cardiovascular risk already starts when kidney function deteriorates below 75 mL/min [2]. The main reason for morbidity for these patients are cardiovascular and infections [2,3]. The choice and extent of dialysis did not alter the outcome of infections in the HEMO study [3]. The reason for morbidity is multifactorial although focused on malnutrition and inflammatory processes, in parallel to atherosclerosis. This triad has been named the MIA-syndrome [4]. Thereby malnutrition is found in 50% of HD patients [5,6]. During progressive kidney failure retention of various solutes occur that are denominated uremic toxins [7,8]. The toxic effect of these substances has not been clarified for many of them. Key functions for a healthy body are an adequate nutrition. The malnutrition present in uremic patients may be due to loss of appetite but also poor absorption from a dysfunctional intestine, increased catabolism but also metabolic dysfunction. Thereby for carbohydrates, degraded into glucose as nutrient, insulin is a key hormone for metabolism. Notable is a progressive insulin resistance in parallel with uraemia [9].

### 1.1. Energy Supply by Triglyceride Breakdown

Fat constitutes approximately 40% of the energy resources in our meals, depending on type of diet. The uptake of fat is mainly as chylomicrons through the intestine. Lipase from salivary glands and pancreas contribute to intestinal break down of fat. Once absorbed into the circulation, the metabolism of fat is mainly through hydrolysis of triglycerides into free fatty acids. This is managed mainly through degradation by the water-soluble hepatic lipase (HL) and by endothelial lipoprotein lipase (LPL), present on the endothelial surface in capillaries in the area of skeletal muscles, heart and adipose tissue [10,11]. Lipoprotein lipase requires Apo-C-II as a cofactor [12,13]. The free fatty acids [14] achieved by the lipase activity will be the main stores of energy derived from fat for the body. Free fatty acids are bound to albumin in plasma and transported to where they are needed or restructured in the fat cells into triglycerides. While LPL mainly metabolises chylomicrons and very low-density lipoproteins the hepatic lipase mainly metabolises intermediate density lipoproteins and low-density lipoproteins. Another lipase, the endothelial lipase, is suggested to be the predominant enzyme responsible for lipolytic catabolism of high-density lipoprotein in hemodialyzed patients [15]. Besides SN1-lipases, several SN2-lipases are expected to play an important role in uremic subjects, these class of enzymes are in part acute phase proteins that might play an important role under inflammatory conditions. Increased type IIA secretory phospholipase A(2) expression contributes to oxidative stress in end-stage renal disease [16].

### 1.2. Interaction of Heparin to Lipase Attachment

To prevent the haemodialysis circuit for clotting in most centres heparin is used [17]. Besides anticoagulation heparin may also exert some anti-inflammatory actions although these probably are limited [18]. However, by administering heparin intravenously the lipoprotein lipase enzymes will to a large extent be detached from their site on the endothelial surface [19,20]. The release of the HL and LPL from its bindings sites causes an immediate increase of the enzymes in the plasma [20] and in parallel an increased degradation of triglycerides into free fatty acid within the plasma [19,20]. However, the loss of endothelial enzyme activity takes several hours to recover, since the free circulating enzymes are rapidly degraded by the liver [21,22]. Thereby the body pool of lipase is extremely depleted within 90 min after a bolus of heparin, dalteparin or tinzaparin [21]. Notable is that there are differences in effect of the various low molecular weight heparins (LMWH) and unfractionated heparin (UFH) [19,21,23]. Although the peak varies the pool of HL and LPL are similarly depleted, indicating a faster uptake and degradation of the enzyme being in a complex with, e.g., dalteparin than with UFH [21]. The hepatic lipase pool seems to recover faster than the LPL pool [21]. When the lipase pool is depleted the triglycerides will rise significantly in plasma for more than 8 h [21].

### 1.3. Lipases, Uremic and Haemodialysis Conditions

**Figure 1:**
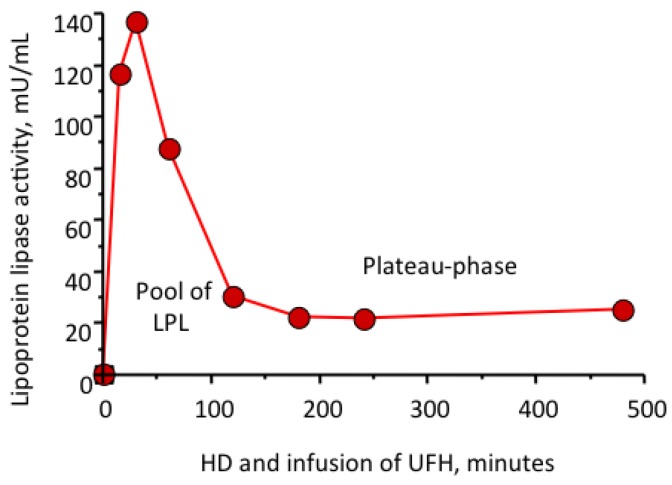
Plasma lipoprotein lipase distribution in a haemodialysis patient after a bolus and infusion of unfractionated heparin (UFH). The figure shows a peak of lipoprotein lipase (LPL) at 30 min and a reduction despite continuous heparin infusion. The area under the curve from start to 120 min represents the pool of LPL. The following plateau represents the capacity of regeneration of LPL.

In uremic patients the triglyceride/cholesterol ration is increased [24,25,26]. The dyslipidaemia is considered to be related to disturbed function of the lipoprotein lipase [6]. The lipase activity is lowered in uremic subjects as well as in HD patients, measured either by lipolysis activity [27,28] or hepatic lipase [29] and plasma lipoprotein lipase activity measured by release of FFA [24] or directly [30]. When analyzing the area under the curve of the pool there is only 50% as much in the HD patients *versus* the controls [21]. Again there is a difference in the levels when administering LMWH *versus* UFH [21,28,30,31]. The difference is similar in the HD patients between the drugs as in control subjects although the HD patients have less than half as much in their pool [32], again an indicator of faster degradation of the dalteparin-LPL complex [32]. The enzyme activity levels off at a plateau phase in plasma where the activity is less than 10% of the maximum activity [32] as seen in Figure 1. Tinzaparin was shown to have an intermittent effect compared to dalteparin and UFH [33].

Notable is the repeated administration of LMWH or UFH and exhaustion of the lipase pools during at least 8 h each haemodialysis, at least 3 times/week. Besides the temporary loss of lipase pool, the enzyme pool is not progressively impaired over time, neither by UFH nor by tinzaparin [33]. However, tinzaparin resulted in a worse triglyceride profile during HD than UFH [33].

### 1.4. Cofactors and Inhibitors to Lipases

In plasma, the presence of the cofactor apoC-II is counteracted by various inhibitors such as apoA-I and apoC-III [34,35]. A more pronounced inhibitory activity has been seen in plasma from uremic subjects [30,35] while no such negative effect was found in the ultrafiltrate [35]. Animal studies revealed that angiopoietin-like protein 3 and 4 (ANGPTL) are inhibitors of the lipoprotein lipase system. The ANGPTL4 interacts with LPL and causes dissociation of active LPL dimers to inactive monomers [36]. A similar mechanism of inactivation has been proposed for ANGPTL3 [37]. It is notable that the plasma concentrations in HD patients are increased compared to ANGPTL4 [38,39] while there are conflicting results regarding ANGPTL3 with either increased [39] or reduced levels [40]. The reason for this difference is not clear. One possibility is a different reactivity of the antibodies used and the complex pattern of molecular forms of the ANGPTLs in plasma. High flux HD, but not low flux, will reduce the level of ANGPTL4 but not ANGPTL3 in plasma [39].

### 1.5. Measures in Dialysis to Counteract Release of Lipases

What options may help to limit the loss of lipase pool during dialysis? Dialysis using citrate in the dialysate was shown not to cause a release of the LPL from its binding sites [39]. However, this type of dialysis resulted in frequent clotting problems when not adding UFH or LMWH [41]. The use of regional citrate anticoagulation has to be further explored in this setting. However, it is not clear to what extent the system is safe, not causing arrhythmia and other side effects [17,42]. This has to be further clarified.

In patients on peritoneal dialysis there is no need of intermittent doses of LMWH or UFH, and thereby no release of LPL and HL from its bindings sites. However, the uremic toxins result in a similar lowered base line level of the lipases in PD as in HD patients, indicating severe metabolic disturbance. The loss of lipases during conventional haemodialysis results in a process of repeated metabolic starvation that does not appear in PD [43]. In HD, this loss of lipases by the anticoagulation at each dialysis process results in malnutrition that will also afflict the cell functions negatively by a lack of free fatty acids for energy supply. Such lack may contribute to increased risk for inflammation, infections and endothelial dysfunction including atherosclerosis, all ending up in the MIA syndrome. Here, PD patients are favoured to maintain lipases and thereby to a lesser extent will suffer from malnutrition. Since addition of glucose is present in PD solutions there may even be an increase in weight gain, a benefit in malnutrition but a disadvantage if the patient is obese.

## 2. Conclusions

In summary, these studies show that uraemia results in a decreased activity of lipases that contribute to a large part of our energy resources. A combined problem is present, firstly, resulting in lowered production of lipases and secondly, by repeated degradation of the lipase pool during conventional anticoagulation for standard HD. The presence of numerous inhibitors adds to this problem. The results of intermittent lipase loss will also result in a lack of free fatty acids over time and thereby shift the patient temporarily into an intermittent metabolic starvation of the patients. An option for malnourished patients having HD could be to change them for peritoneal dialysis. Further studies may clarify if citrate dialysis favours the energy balance of HD patients in the long run.

## References

[1] Schon S., Ekberg H., Wikstrom B., Oden A., Ahlmen J. (2004). Renal replacement therapy in sweden. Scand. J. Urol. Nephrol..

[2] Vanholder R., Massy Z., Argiles A., Spasovski G., Verbeke F., Lameire N. (2005). Chronic kidney disease as cause of cardiovascular morbidity and mortality. Nephrol. Dial. Transplant..

[3] Allon M., Depner T.A., Radeva M., Bailey J., Beddhu S., Butterly D., Coyne D.W., Gassman J.J., Kaufman A.M., Kaysen G.A. (2003). Impact of dialysis dose and membrane on infection-related hospitalization and death: Results of the hemo study. J. Am. Soc. Nephrol..

[4] Stenvinkel P., Heimburger O., Lindholm B., Kaysen G.A., Bergstrom J. (2000). Are there two types of malnutrition in chronic renal failure? Evidence for relationships between malnutrition, inflammation and atherosclerosis (mia syndrome). Nephrol. Dial. Transplant..

[5] Qureshi A.R., Alvestrand A., Danielsson A., Divino-Filho J.C., Gutierrez A., Lindholm B., Bergstrom J. (1998). Factors predicting malnutrition in hemodialysis patients: A cross-sectional study. Kidney Int..

[6] Carrero J.J., Stenvinkel P., Cuppari L., Ikizler T.A., Kalantar-Zadeh K., Kaysen G., Mitch W.E., Price S.R., Wanner C., Wang A.Y. (2013). Etiology of the protein-energy wasting syndrome in chronic kidney disease: A consensus statement from the international society of renal nutrition and metabolism (isrnm). J. Ren. Nutr..

[7] Vanholder R., Argiles A., Baurmeister U., Brunet P., Clark W., Cohen G., De Deyn P.P., Deppisch R., Descamps-Latscha B., Henle T. (2001). Uremic toxicity: Present state of the art. Int. J. Artif. Organs.

[8] Vanholder R., De Smet R., Glorieux G., Argiles A., Baurmeister U., Brunet P., Clark W., Cohen G., De Deyn P.P., Deppisch R. (2003). Review on uremic toxins: Classification, concentration, and interindividual variability. Kidney Int..

[9] Rigalleau V., Gin H. (2005). Carbohydrate metabolism in uraemia. Curr. Opin. Clin. Nutr. Metab. Care.

[10] Wang H., Eckel R.H. (2009). Lipoprotein lipase: From gene to obesity. Am. J. Physiol. Endocrinol. Metab..

[11] Stegmayr B., Olivecrona T., Olivecrona G. (2009). Lipoprotein lipase disturbances induced by uremia and hemodialysis. Semin. Dial..

[12] Kinnunen P.K., Jackson R.L., Smith L.C., Gotto A.M., Sparrow J.T. (1977). Activation of lipoprotein lipase by native and synthetic fragments of human plasma apolipoprotein c-ii. Proc. Natl. Acad. Sci. USA.

[13] Kim S.Y., Park S.M., Lee S.T. (2006). Apolipoprotein c-ii is a novel substrate for matrix metalloproteinases. Biochem. Biophys. Res. Commun..

[14] Pianta T.J., Horvath A.R., Ellis V.M., Leonetti R., Moffat C., Josland E.A., Brown M.A. (2012). Cardiac high-sensitivity troponin t measurement: A layer of complexity in managing haemodialysis patients. Nephrology.

[15] Miksztowicz V., McCoy M.G., Schreier L., Cacciagiu L., Elbert A., Gonzalez A.I., Billheimer J., Eacho P., Rader D.J., Berg G. (2012). Endothelial lipase activity predicts high-density lipoprotein catabolism in hemodialysis: Novel phospholipase assay in postheparin human plasma. Arterioscler. Thromb. Vasc. Biol..

[16] Van der Giet M., Tolle M., Pratico D., Lufft V., Schuchardt M., Horl M.P., Zidek W., Tietge U.J. (2010). Increased type iia secretory phospholipase a(2) expression contributes to oxidative stress in end-stage renal disease. J. Mol. Med..

[17] Cronin R.E., Reilly R.F. (2010). Unfractionated heparin for hemodialysis: Still the best option. Semin. Dial..

[18] Cornet A.D., Smit E.G., Beishuizen A., Groeneveld A.B. (2007). The role of heparin and allied compounds in the treatment of sepsis. Thromb. Haemost..

[19] Persson E., Nordenstrom J., Nilsson-Ehle P., Hagenfeldt L. (1985). Lipolytic and anticoagulant activities of a low molecular weight fragment of heparin. Eur. J. Clin. Invest..

[20] Nasstrom B., Olivecrona G., Olivecrona T., Stegmayr B.G. (2001). Lipoprotein lipase during continuous heparin infusion: Tissue stores become partially depleted. J. Lab. Clin. Med..

[21] Nasstrom B., Olivecrona G., Olivecrona T., Stegmayr B.G. (2003). Lipoprotein lipase during heparin infusion: Lower activity in hemodialysis patients. Scand. J. Clin Lab. Invest..

[22] Olivecrona T., Olivecrona G., Betteridge D.J., Illingworth D.R., Shepard J. (1999). Lipoprotein and hepatic lipases in lipoprotein metabolism. Lipoproteins in Health and Disease.

[23] Schrader J., Stibbe W., Armstrong V.W., Kandt M., Muche R., Kostering H., Seidel D., Scheler F. (1988). Comparison of low molecular weight heparin to standard heparin in hemodialysis/hemofiltration. Kidney Int..

[24] Chan M.K., Persaud J., Varghese Z., Moorhead J.F. (1984). Pathogenic roles of post-heparin lipases in lipid abnormalities in hemodialysis patients. Kidney Int..

[25] Attman P.O., Alaupovic P., Tavella M., Knight-Gibson C. (1996). Abnormal lipid and apolipoprotein composition of major lipoprotein density classes in patients with chronic renal failure. Nephrol. Dial. Transplant..

[26] Attman P.O., Samuelsson O., Alaupovic P. (2011). The effect of decreasing renal function on lipoprotein profiles. Nephrol. Dial. Transplant..

[27] Bagdade J.D., Yee E., Wilson D.E., Shafrir E. (1978). Hyperlipidemia in renal failure: Studies of plasma lipoproteins, hepatic triglyceride production, and tissue lipoprotein lipase in a chronically uremic rat moedl. J. Lab. Clin Med..

[28] Schrader J., Andersson L.O., Armstrong V.W., Kundt M., Stibbe W., Scheler F. (1990). Lipolytic effects of heparin and low molecular weight heparin and their importance in hemodialysis. Semin. Thromb. Hemost..

[29] Oi K., Hirano T., Sakai S., Kawaguchi Y., Hosoya T. (1999). Role of hepatic lipase in intermediate-density lipoprotein and small, dense low-density lipoprotein formation in hemodialysis patients. Kidney Int. Suppl..

[30] Arnadottir M., Kurkus J., Nilsson-Ehle P. (1994). Different types of heparin in haemodialysis: Long-term effects on post-heparin lipases. Scand. J. Clin. Lab. Invest..

[31] Nasstrom B., Stegmayr B., Gupta J., Olivecrona G., Olivecrona T. (2005). A single bolus of a low molecular weight heparin to patients on haemodialysis depletes lipoprotein lipase stores and retards triglyceride clearing. Nephrol. Dial. Transplant..

[32] Nasstrom B., Stegmayr B., Olivecrona G., Olivecrona T. (2004). Lipoprotein lipase in hemodialysis patients: Indications that low molecular weight heparin depletes functional stores, despite low plasma levels of the enzyme. BMC Nephrol..

[33] Mahmood D., Grubbstrom M., Lundberg L.D., Olivecrona G., Olivecrona T., Stegmayr B.G. (2010). Lipoprotein lipase responds similarly to tinzaparin as to conventional heparin during hemodialysis. BMC Nephrol..

[34] Kaysen G.A. (1994). Hyperlipidemia of chronic renal failure. Blood Purif..

[35] Cheung A.K., Parker C.J., Ren K., Iverius P.H. (1996). Increased lipase inhibition in uremia: Identification of pre-beta-hdl as a major inhibitor in normal and uremic plasma. Kidney Int..

[36] Sukonina V., Lookene A., Olivecrona T., Olivecrona G. (2006). Angiopoietin-like protein 4 converts lipoprotein lipase to inactive monomers and modulates lipase activity in adipose tissue. Proc. Natl. Acad. Sci. USA.

[37] Lee E.C., Desai U., Gololobov G., Hong S., Feng X., Yu X.C., Gay J., Wilganowski N., Gao C., Du L.L. (2009). Identification of a new functional domain in angiopoietin-like 3 (angptl3) and angiopoietin-like 4 (angptl4) involved in binding and inhibition of lipoprotein lipase (lpl). J. Biol. Chem..

[38] Baranowski T., Kralisch S., Bachmann A., Lossner U., Kratzsch J., Bluher M., Stumvoll M., Fasshauer M. (2011). Serum levels of the adipokine fasting-induced adipose factor/angiopoietin-like protein 4 depend on renal function. Horm. Metab. Res..

[39] Mahmood D., Makoveichuk E., Nilsson S., Olivecrona G., Stegmayr B. (2014). Response of angiopoietin-like proteins 3 and 4 to haemodialysis. Int. J. Artif. Organs.

[40] Shoji T., Hatsuda S., Tsuchikura S., Kimoto E., Kakiya R., Tahara H., Koyama H., Emoto M., Tabata T., Nishizawa Y. (2009). Plasma angiopoietin-like protein 3 (angptl3) concentration is associated with uremic dyslipidemia. Atherosclerosis.

[41] Stegmayr B.G., Jonsson P., Mahmood D. (2013). A significant proportion of patients treated with citrate containing dialysate need additional anticoagulation. Int. J. Artif. Organs.

[42] Buturovic-Ponikvar J., Gubensek J., Ponikvar R. (2008). Citrate anticoagulation for postdilutional online hemodiafiltration with calcium-containing dialysate and infusate: Significant clotting in the venous bubble trap. Int. J. Artif. Organs.

[43] Mahmood D., Nilsson S., Olivecrona G., Stegmayr B. (2014). Lipoprotein lipase activity is favoured by peritoneal dialysis compared to hemodialysis. Scand. J. Clin. Lab. Invest..

